# Compared with matched controls, patients with postoperative atrial fibrillation (POAF) have increased long-term AF after CABG, and POAF is further associated with increased ischemic stroke, heart failure and mortality even after adjustment for AF

**DOI:** 10.1007/s00392-020-01614-z

**Published:** 2020-02-08

**Authors:** Emma Thorén, Mona-Lisa Wernroth, Christina Christersson, Karl-Henrik Grinnemo, Lena Jidéus, Elisabeth Ståhle

**Affiliations:** 1 Department of Surgical Sciences, Cardiothoracic Surgery, Uppsala University, Uppsala, Sweden; 2 Department of Medical Sciences, Molecular Epidemiology and Science for Life Laboratory, and Uppsala Clinical Research Center (UCR), Uppsala University, Uppsala, Sweden; 3 Department of Medical Sciences, Cardiology, Uppsala University, Uppsala, Sweden

**Keywords:** Coronary artery bypass graft surgery, Atrial fibrillation, Stroke, Heart failure, Mortality

## Abstract

**Objective:**

To analyze (1) associations between postoperative atrial fibrillation (POAF) after CABG and long-term cardiovascular outcome, (2) whether associations were influenced by AF during follow-up, and (3) if morbidities associated with POAF contribute to mortality.

**Methods:**

An observational cohort study of 7145 in-hospital survivors after isolated CABG (1996–2012), with preoperative sinus rhythm and without AF history. Incidence of AF was compared with matched controls. Time-updated covariates were used to adjust for POAF-related morbidities during follow-up, including AF.

**Results:**

Thirty-one percent of patients developed POAF. Median follow-up was 9.8 years. POAF patients had increased AF compared with matched controls (HR 3.03; 95% CI 2.66–3.49), while AF occurrence in non-POAF patients was similar to controls (1.00; 0.89–1.13). The observed AF increase among POAF patients compared with controls persisted over time (> 10 years 2.73; 2.13–3.51). Conversely, the non-POAF cohort showed no AF increase beyond the first postoperative year. Further, POAF was associated with long-term AF (adjusted HR 3.20; 95% CI 2.73–3.76), ischemic stroke (1.23; 1.06–1.42), heart failure (1.44; 1.27–1.63), overall mortality (1.21; 1.11–1.32), cardiac mortality (1.35; 1.18–1.54), and cerebrovascular mortality (1.54; 1.17–2.02). These associations remained after adjustment for AF during follow-up. Adjustment for other POAF-associated morbidities weakened the association between POAF and overall mortality, which became non-significant.

**Conclusions:**

Patients with POAF after CABG had three times the incidence of long-term AF compared with both non-POAF patients and matched controls. POAF was associated with long-term ischemic stroke, heart failure, and corresponding mortality even after adjustment for AF during follow-up. The increased overall mortality was partly explained by morbidities associated with POAF.

**Graphic abstract:**

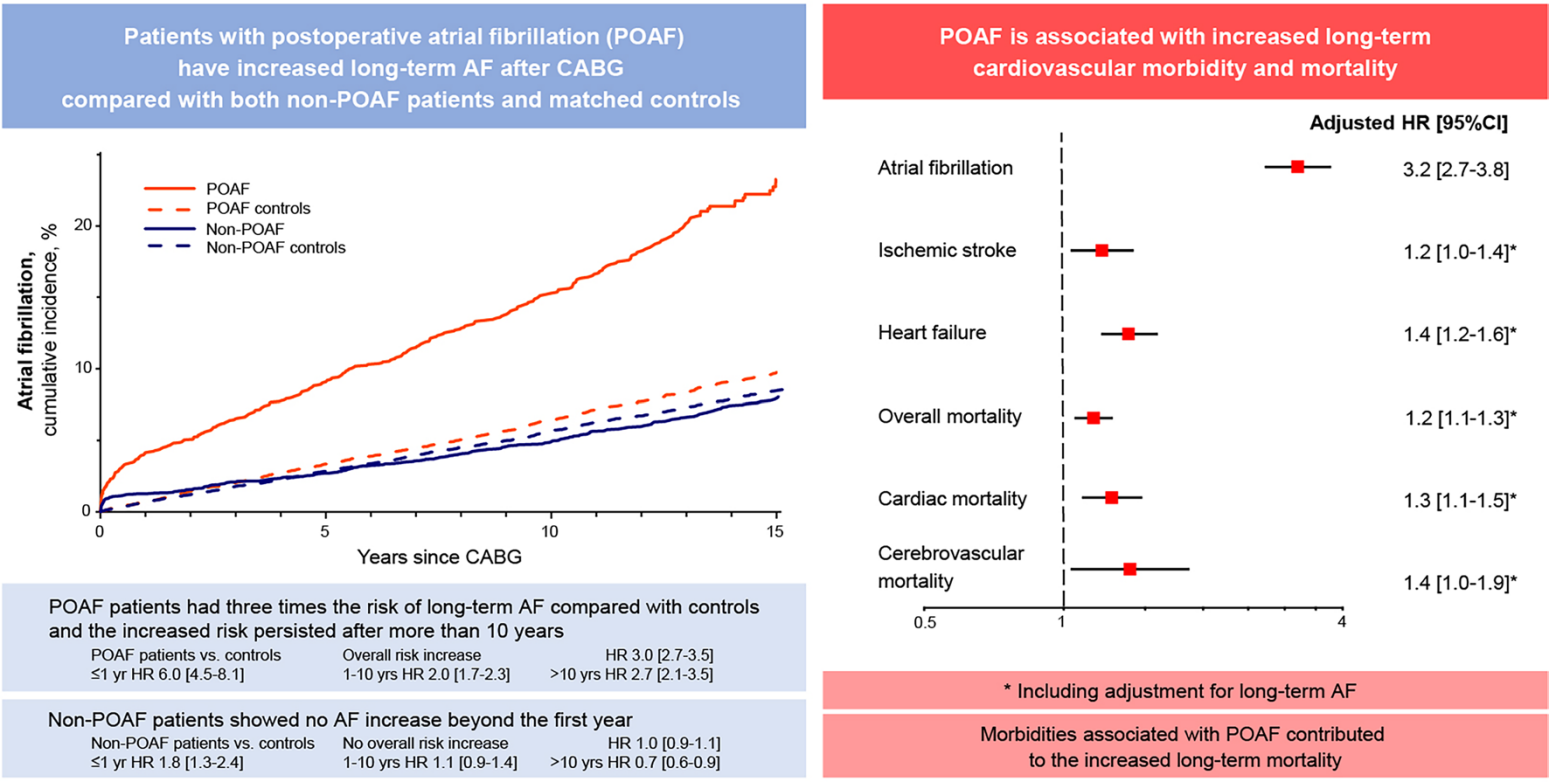

**Electronic supplementary material:**

The online version of this article (10.1007/s00392-020-01614-z) contains supplementary material, which is available to authorized users.

## Introduction

New-onset postoperative atrial fibrillation (POAF) affects about one-third of all patients that undergo coronary artery bypass grafting (CABG) [1], which makes long-term consequences highly relevant. However, except for recurrent atrial fibrillation (AF) [2–4] and mortality [2, 5, 6], POAF has not been associated with long-term cardiovascular outcome after CABG, including ischemic stroke and heart failure*,* and never with any corresponding biological mechanisms. Ischemic stroke, which is the main concern for patients with primary AF [7], has only been associated with POAF after cardiac surgery in a subgroup analysis of a single study, based on all patients hospitalized for surgery [8].

Cardiac surgery patients have a number of characteristics in common, e.g. pericardiotomy and the use of extracorporeal circulation, which are both associated with increased systemic inflammation and increased risk of POAF [9–11]. Despite similarities, the cardiac surgery cohort is still a heterogeneous group. Patients belonging to the two largest subgroups, CABG and valve procedures, differ significantly from each other, and within the valve group there are also substantial differences, e.g. between patients with aortic valve stenosis versus mitral regurgitation. Without relevant sensitivity or interaction analyses, results from such a heterogeneous group cannot be directly applied to CABG patients [8]. In contrast to patients with valvular heart disease, a CABG cohort is homogenous, and results regarding associations between POAF and cardiovascular outcome derived from that cohort are thus generalizable to the large and important CABG population [12].

For primary AF, associations with long-term cardiovascular outcome [13, 14] as well as underlying mechanisms are well recognized [15, 16]. There are established mechanisms as to how AF contributes to an increased risk of thromboembolism, specifically ischemic stroke [15]. Irregular heart rhythm causes stasis in the left atrium, resulting in blood flow abnormalities that activate the coagulation cascade [15]. Furthermore, AF promotes different levels of vessel wall damage [15]. Regarding AF and heart failure, it has been suggested that they are different expressions of the same underlying systemic disease, where inflammation is significantly involved in both conditions and in many of their shared comorbidities [16–19]. In short, in the context of primary AF there are several identified mechanisms regarding associations with outcome. However, in the context of POAF, comparable studies are sparse, and no corresponding underlying mechanisms have been proven valid [20, 21]. In lack of relevant knowledge about mechanisms that explain associations between POAF in relation to CABG and cardiovascular outcome, we hypothesized that the same mechanisms as for primary AF would be involved. Still, it is important to recognize that, in lack of a verified mechanism, any potential associations between POAF and outcome should not be considered as evidence for a causal relationship. POAF may also constitute a marker for more advanced disease, e.g. pathologies engaging the atrial wall and/or ventricular myocardium that are due to long-standing hypoxia in CABG patients, which in turn are associated with an impaired prognosis.

Based on prior studies, patients with POAF can be expected to have a higher incidence of AF during follow-up [2–4]. Consequently, any associations between POAF and outcome might also be confounded by the increased occurrence of AF during follow-up.

The purpose of this study was to examine the impact of POAF on long-term cardiovascular morbidity and mortality. Incidences of AF in patients with and without POAF were compared to those observed in matched controls from the general population. We aimed to analyze (1) potential associations, (2) whether associations were influenced by AF during follow-up, and (3) if morbidities associated with POAF contribute to mortality.

## Methods

### Study design and population

In this observational cohort study, all patients who underwent primary isolated CABG at the Department of Cardiothoracic Surgery, Uppsala University Hospital, Sweden, from January 1996 through December 2012 (*N* = 8074), were considered. Patients who died in relation to the index surgery (*n* = 23) or during hospitalization in connection with the index surgery (*n* = 168) were excluded, as well as non-Swedish citizens (*n* = 151). After excluding patients that were not in sinus rhythm at admission (*n* = 334) or had a history of AF (i.e. hospital admissions for AF prior to the index surgery, *n* = 253), the final cohort included 7145 patients.

Routinely, a twelve-lead electrocardiogram (ECG) was taken at admission and on the fourth postoperative day, and patients were monitored by five-lead telemetry until the third postoperative day. Additional ECGs and prolonged telemetry were initiated for patients with suspected arrhythmias. POAF was defined as an episode of AF requiring intervention that developed during hospitalization in relation to the index surgery. If indicated, patients with POAF were treated with amiodarone or sotalol and/or by cardioversion, with the strategy that all patients should be discharged in sinus rhythm [6]. Oral anticoagulants were not routinely prescribed to patients with POAF.

The study was approved by the Regional Ethical Review Board in Uppsala (approval # 2010/453) and complies with the Declaration of Helsinki.

### Data collection

All patients were prospectively registered in the department’s database. Patients were followed from hospital discharge until death or until end of follow-up (December 31, 2013) through linkage with the Cause of Death Registry (CDR) and with the National Patient Registry (NPR), both held by the Swedish National Board of Health and Welfare. The NPR contains data on all hospital admissions in Sweden since 1987, with a validity of diagnoses of cardiovascular disease of approximately 95% [22]. Data concerning medical history were obtained for a 5-year period prior to the index surgery (Online Resource, Table S1). For each CABG patient (health status = patient), four to five controls (mean 4.99 ± 0.09) (health status = control) were identified in the Total Population Register. Controls were matched for age, gender and county and constituted a control group regarding the occurrence of AF during follow-up. Based on POAF status, patients and corresponding controls were subdivided into a POAF and a non-POAF cohort, respectively.

### Outcomes

Overall mortality was defined as death from any cause. Cause-specific mortality was defined as a cardiac or cerebrovascular diagnosis as underlying cause of death in the CDR. Long-term morbidity events were defined as AF, ischemic stroke, heart failure, non-cerebral thromboembolism, hemorrhagic stroke, or non-cerebral bleeding during follow-up, as primary diagnosis in the NPR or underlying cause of death in the CDR (Online Resource, Table S2).

### Statistical analysis

Baseline characteristics and surgical variables were compared with independent sample t-tests, Wilcoxon-Mann–Whitney *U* tests, or Chi-square tests, as appropriate. For morbidity events, cumulative incidence curves (CIF) were constructed, treating death from other causes as a competing risk. CIF curves for AF were also constructed for matched POAF and non-POAF controls. To estimate the burden of AF during follow-up with regard to POAF, incidence rates were calculated based on all episodes of AF during follow-up in each individual patient and the corresponding matched controls, presented as number of events per 1000 person-years (py) with 95% CI. Comparisons were made between POAF and non-POAF patients, and between patients and controls with Wilcoxon two-sample rank-sum tests based on individual incidence rates.

Cox proportional hazards models were used to compare time to outcome events between patients with and without POAF. Potential confounders were selected based on prior knowledge. The following variables were adjusted for: age, gender, hypertension, medical history of ischemic stroke, heart failure, non-cerebral thromboembolism, hemorrhagic stroke, non-cerebral bleeding, and diabetes, number of diseased coronary vessels, left main stenosis, left ventricular function [preoperative ejection fraction normal (> 50%), reduced (30–50%), or poor (< 30%)], time of surgery, use of the internal mammary artery (IMA), and aortic cross-clamp time.

Results are presented as adjusted HR with 95% CI. The baseline hazard function was stratified by year of surgery. The proportional hazard assumption was tested using Schoenfeld residuals and was found to be valid.

AF during follow-up was adjusted for by including a time-updated covariate in Cox models for overall and cause-specific mortality, and significant morbidities. Morbidity events during follow-up significantly associated with POAF were included as time-updated covariates in Cox models regarding overall mortality.

Overall survival for patients with and without POAF was compared with the log-rank test and presented as Kaplan–Meier survival curves.

Interaction between POAF and health status (patient versus control) regarding AF during follow-up was tested by the inclusion of a multiplicative interaction term in an otherwise unadjusted Cox analysis. The persistence of the effect of health status on AF during follow-up over time was investigated by allowing effects to change between time intervals, i.e. ≤ 1 year, 1–10 years, and > 10 years after surgery, for the POAF and non-POAF cohort, respectively. Interactions between health status and time after surgery were tested by the inclusion of a multiplicative interaction term.

Interactions between POAF and gender were tested by the inclusion of a multiplicative interaction term. Regarding POAF-associated outcome events, interactions between POAF and AF during follow-up, and POAF and postoperative course, were examined by the inclusion of a multiplicative interaction term. An uncomplicated course was defined as no sternal wound infection, stroke, heart failure (need for inotropic drugs and/or intra-aortic balloon pump or left ventricular assist device), myocardial infarction (elevated cardiac markers), reoperation, or renal dysfunction (postoperative S-creatinine > 200 µgL^−1^) in relation to the index surgery (*n* = 5695).

A two-tailed *P* value < 0.05 was considered statistically significant. All statistical analyses were performed using SAS version 9.4 (SAS Institute Inc., Cary, NC, USA).

## Results

Median follow-up time was 9.8 years (IQR 6.1–13.7). During this time, 30.6% of patients (2183 of 7145) developed POAF after CABG. Patients with POAF were older, but without other relevant differences from patients without POAF (Table 1). The majority of the surgical procedures (96.7%) were performed with the use of cardiopulmonary bypass (on-pump cross-clamp technique).

**Table 1:** Baseline characteristics and surgical variables according to POAF

Variable	POAF	Non-POAF	*P* value
	*n* = 2183 (30.6%)	*n* = 4962 (69.4%)	
Age, years, mean ± SD	68.9 ± 7.7	65.1 ± 9.1	< 0.001
Male gender, *n (%)*	1724 (79.0)	3829 (77.2)	0.09
Hypertension, *n (%)*	1168 (53.5)	2575 (51.9)	0.22
Medical history within 5 years of surgery*, n (%)*			
Ischemic stroke	73 (3.3)	145 (2.9)	0.34
Heart failure	149 (6.8)	324 (6.5)	0.64
Non-cerebral thromboembolism	57 (2.6)	96 (1.9)	0.07
Hemorrhagic stroke	4 (0.2)	18 (0.4)	0.21
Non-cerebral bleeding	58 (2.7)	94 (1.9)	0.04
Diabetes	314 (14.4)	728 (14.7)	0.75
Diseased coronary vessels ≥ 3*, n (%)*	1634 (74.9)	3694 (74.4)	0.71
Left main stenosis, *n (%)*	758 (34.7)	1653 (33.3)	0.25
Left ventricular function, *n (%)*			
Normal (> 50%)	1507 (69.0)	3503 (70.6)	0.20
Reduced (30–50%)	572 (26.2)	1265 (25.5)	
Poor (< 30%)	104 (4.8)	194 (3.9)	
Year of surgery, *n (%)*			
1996–1999	838 (38.4)	2043 (41.2)	< 0.001
2000–2004	764 (35.0)	1469 (29.6)	
2005–2012	581 (26.6)	1450 (29.2)	
Use of IMA, *n (%)*	2012 (92.2)	4561 (91.9)	0.72
Aortic cross-clamp time, median (IQR)^a^	43 (35–53)	43 (34–53)	0.07

*IMA* internal mammary artery, *POAF* postoperative atrial fibrillation

^a^Variable available for on-pump CABG (*n* = 6907)

### AF during follow-up

**Fig. 1:**
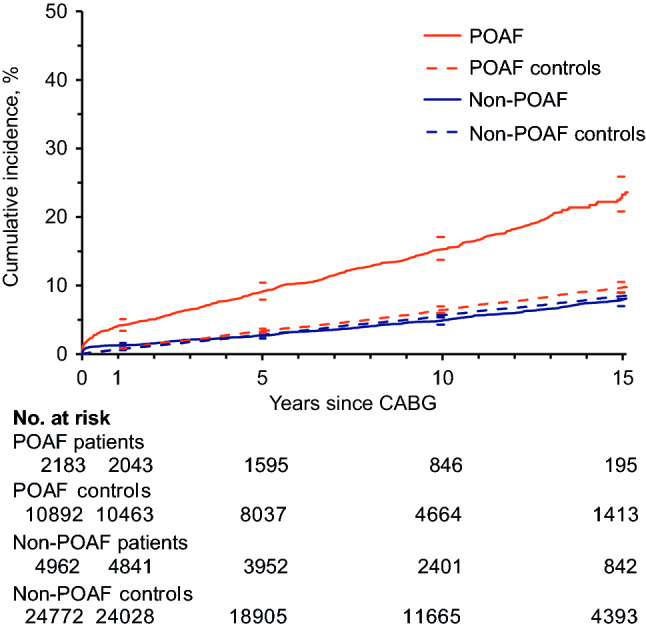
Cumulative incidence of atrial fibrillation during follow-up for patients with and without POAF after CABG and their corresponding matched controls, with 95% CI. *CABG* coronary artery bypass grafting; *POAF* postoperative atrial fibrillation

The cumulative incidence of AF during follow-up at 10 years was 15.3% among POAF patients versus 6.4% among matched controls (Fig. 1). Corresponding figures regarding non-POAF patients were 4.9% versus 5.7%. Patients with POAF had higher incidence rates of AF during follow-up (32.3 events per 1000 py; 95% CI 30.0–34.9) both compared with non-POAF patients (9.0 events per 1000 py; 95% CI 8.2–9.9; *P* < 0.001), and compared with matched controls (13.9 events per 1000 py; 95% CI 13.2–14.7; *P* < 0.001). However, there was no difference between non-POAF patients and their matched controls (9.0 versus 12.6 events per 1000 py; 95% CI 12.2–13.1; *P* = 0.43).

POAF was associated with AF during follow-up (HR 3.20; 95% CI 2.73–3.76; Table 2).

**Table 2:** Associations between POAF and outcome events

	Number with event (%)		Univariable Cox Model		Multivariable Cox Model	
Outcome event	POAF *n* = 2183	Non-POAF *n* = 4962	HR (95% CI)	*P* value	HR (95% CI)^a^	*P* value
Atrial fibrillation	353 (16.2)	282 (5.7)	3.31 (2.83; 3.87)	< 0.001	3.20 (2.73; 3.76)	< 0.001
Ischemic stroke	282 (12.9)	490 (9.9)	1.43 (1.24; 1.66)	< 0.001	1.23 (1.06; 1.42)	0.008
Heart failure	416 (19.1)	644 (13.0)	1.65 (1.46; 1.87)	< 0.001	1.44 (1.27; 1.63)	< 0.001
Non-cerebral thromboembolism	194 (8.9)	368 (7.4)	1.31 (1.10; 1.56)	0.002	1.14 (0.95; 1.36)	0.15
Hemorrhagic stroke	61(2.8)	101 (2.0)	1.50 (1.09; 2.06)	0.01	1.25 (0.90; 1.73)	0.18
Non-cerebral bleeding	163 (7.5)	312 (6.3)	1.28 (1.06; 1.55)	0.01	1.13 (0.93; 1.37)	0.23
Overall mortality	906 (41.5)	1550 (31.2)	1.49 (1.37; 1.62)	< 0.001	1.21 (1.11; 1.32)	< 0.001
Cardiac mortality	390 (17.9)	606 (12.2)	1.65 (1.46; 1.88)	< 0.001	1.35 (1.18; 1.54)	< 0.001
Cerebrovascular mortality	97(4.4)	121 (2.4)	2.04 (1.56; 2.67)	< 0.001	1.54 (1.17; 2.02)	0.002

*HR* hazard ratio, *POAF* postoperative atrial fibrillation

^a^Hazard ratios are adjusted for demographic data, medical history, preoperative and surgical variables (see “Methods”)

There was a significant interaction between POAF and health status revealing that while POAF patients had markedly increased AF compared with corresponding controls (HR 3.03; 95% CI 2.66–3.49), the AF occurrence in patients without POAF was comparable to that observed among their matched controls (HR 1.00; 95% CI 0.89–1.13; *P* < 0.001 for interaction).

The associations between health status and time after surgery regarding AF during follow-up were stronger during the first postoperative year, both in the POAF cohort (HR 6.04; 95% CI 4.53–8.05) and non-POAF cohort (HR 1.80; 95% CI 1.34–2.40). Later during follow-up, comparable associations were weaker. Within the POAF cohort, health status was strongly associated with AF during follow-up, even more than 10 years after surgery (1–10 years: HR 1.97; 95% CI 1.71–2.27; > 10 years: HR 2.73; 95% CI 2.13–3.51; *P* < 0.001 for interaction between health status and time after surgery). However, in the non-POAF cohort there was no significant association between health status and time after surgery beyond the first postoperative year (1–10 years: HR 1.12; 95% CI 0.87–1.44; > 10 years HR 0.72; 95% CI 0.61–0.86; *P* < 0.001 for interaction).

There were no interactions between POAF and gender regarding AF during follow-up (data not shown).

### Morbidity events

**Fig. 2:**
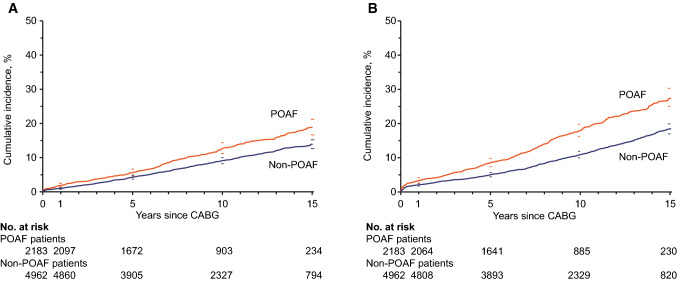
Cumulative incidences of (a) ischemic stroke, and (b) heart failure during follow-up by POAF with 95% CI. *CABG* coronary artery bypass grafting; *POAF* postoperative atrial fibrillation

Out of 7145 patients, 67.2% (4804) did not experience any of the predefined morbidity events during follow-up, 24.5% (1752) had one type of event, 6.9% (495) had two types of events, and 1.3% (94) had three types of events or more. The cumulative incidences of ischemic stroke and heart failure are presented in Fig. 2a, b.

In multivariable Cox analyses, POAF was associated with ischemic stroke (HR 1.23; 95% CI 1.06–1.42) and heart failure (HR 1.44; 95% CI 1.27–1.63) during follow-up (Table 2). There were no independent associations between POAF and non-cerebral thromboembolism, hemorrhagic stroke, or non-cerebral bleeding (Table 2). There were no interactions between POAF and gender regarding morbidity (data not shown).

After adjustment for the occurrence of AF during follow-up, POAF remained associated with ischemic stroke (HR 1.21; 95% CI 1.04–1.41), and there was no interaction between POAF and AF during follow-up (*P* = 0.73 for interaction). POAF also remained associated with heart failure. The association between POAF and HF during follow-up was different in patients without (HR POAF 1.35; 95% CI 1.17–1.56) and with (HR POAF 0.95; 95% CI 0.68–1.33) AF during follow-up (*P* = 0.046 for interaction).

### Overall and cause-specific mortality

**Fig. 3:**
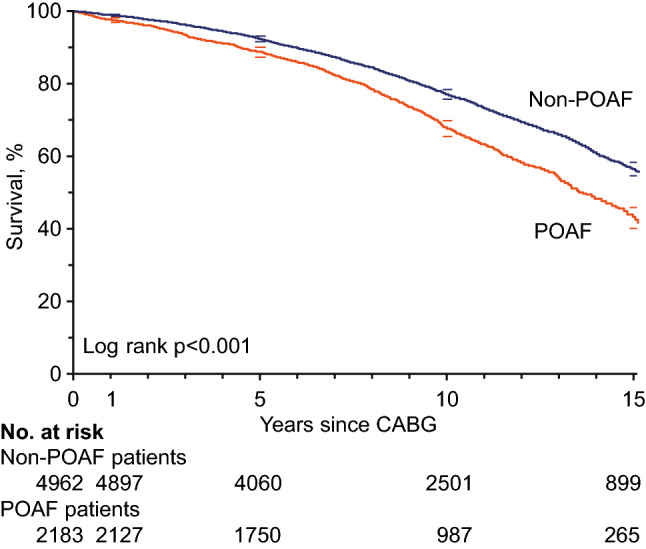
Kaplan–Meier survival curves for overall mortality by POAF with 95% CI**.** *CABG* coronary artery bypass grafting; *POAF* postoperative atrial fibrillation

At the end of follow-up, 2456 patients (34.4%) had died. Out of all deaths, 996 (40.6%) were cardiac mortalities and 218 (8.9%) cerebrovascular mortalities. Patients with POAF had an increased overall mortality, with an absolute difference of 13.5% at 15 years (Fig. 3). Cerebrovascular and cardiac mortality accounted for 75% (7.7% of 10.3%) of the absolute increase in mortality among POAF patients (Table 2). In multivariable Cox analyses, POAF was associated with higher overall mortality (HR 1.21; 95% CI 1.11–1.32; Table 2), cardiac mortality (HR 1.35; 95% CI 1.18–1.54), and cerebrovascular mortality (HR 1.54; 95% CI 1.17–2.02). There were no interactions between POAF and gender regarding overall, cardiac, or cerebrovascular mortality (data not shown).

The occurrence of AF during follow-up did not alter the associations between POAF and mortality, and POAF remained associated with overall (HR 1.16; 95% CI 1.06–1.27; *P* = 0.24 for interaction), cardiac (HR 1.27; 95% CI 1.10–1.47; *P* = 0.29 for interaction), and cerebrovascular mortality (HR 1.39; 95% CI 1.04–1.86; *P* = 0.08 for interaction).

### POAF and postoperative course

There were no interactions between POAF and postoperative course regarding AF during follow-up, ischemic stroke, or heart failure (Online Resource, Table S3). In patients with an uncomplicated postoperative course, POAF was associated with AF during follow-up (HR 3.19; 95% CI, 2.66–3.82), ischemic stroke (HR 1.27; 95% CI 1.07–1.51), and heart failure (HR 1.44; 95% CI 1.24–1.68). Neither were there any interactions between POAF and postoperative course regarding mortality (Online Resource, Table S4). In patients with an uncomplicated postoperative course, POAF was associated with overall (HR 1.19; 95% CI 1.07–1.31), cardiac (HR 1.35; 95% CI 1.16–1.58), and cerebrovascular mortality (HR 1.61; 95% CI 1.17–2.22).

### Morbidity events during follow-up and overall mortality

After adjustment for the occurrence of AF, ischemic stroke and heart failure during follow-up, the association between POAF and overall mortality became weaker (HR 1.06; 95% CI 0.98–1.15) and was no longer statistically significant.

## Discussion

In an isolated CABG cohort, patients with POAF had three times the incidence of long-term AF as compared with both patients without POAF and matched controls in the general population. POAF was further associated with ischemic stroke, heart failure, and mortality (overall, cardiac, and cerebrovascular). These associations were not altered by the occurrence of AF during follow-up, which this study is the first to demonstrate. After adjustment for the occurrence of these morbidities, POAF was no longer associated with overall mortality. Thus, the increased overall mortality was at least partly explained by morbidities associated with POAF.

Patients undergoing cardiac surgery have a much higher incidence of POAF (20–40%) compared with those undergoing non-cardiac surgery (1–3%) [8, 23, 24], and there is a tendency toward an exposure–response relationship, where the incidence of POAF increases as the cardiac surgical approach becomes more invasive [9, 25]. This indicates a certain degree of causality. Moreover, the incidence of POAF has consistently been reported as significantly higher after CABG compared with the much less invasive PCI procedure, for example 18.0% versus 0.1% in a randomized trial with similar patients in both groups [26, 27]. Increased systemic inflammation associated with ECC has been proposed as a potential mechanism regarding the development of POAF after cardiac surgery [9, 10]. This is the first study to show an increased incidence of AF for CABG patients compared with matched controls during the first year after surgery, regardless of POAF status. Altogether, this information strengthens the idea of potential causality, i.e. that cardiac surgery to some extent may cause AF, i.e. POAF.

Furthermore, the current study confirmed that POAF in turn was associated with an increased occurrence of AF during follow-up [2–4]. In fact, patients with POAF had increased incidence of AF not only compared with patients without POAF but also compared with matched, presumably healthy, controls. This information has not been reported previously. Moreover, regarding POAF patients, the increase in AF as compared with controls persisted over time and was valid after more than 10 years of follow-up. On the contrary, the non-POAF cohort showed no increase in AF beyond the first postoperative year. This information supports the idea of potential causality, i.e. that POAF to some extent may cause recurrent AF.

Primary AF leads to recurrent AF through a number of potential mechanisms [28, 29]. There are data that indicate causality, i.e. every episode of AF has the potential to cause electrical, contractile and structural remodeling that in turn promote and/or trigger future AF episodes [28, 29]. It can be proposed that POAF after CABG can cause AF through the same mechanisms. However, based on the current study, we cannot prove or even propose causality. Then again, our results neither refute nor contradict causality as an option. Nevertheless, our study does not rule out that POAF, primarily or partly, occurs in a subset of patients with an inherently greater risk of developing AF in the future.

The present study is the first to demonstrate an association between POAF and long-term risk of ischemic stroke in an isolated CABG cohort. Until now, available results have been few and conflicting regarding POAF and long-term ischemic stroke risk after cardiac surgery [4, 8, 30–32]. Associations identified in previous studies involved broader definitions of outcome, including other thromboembolic events [30, 31], or included a more heterogeneous cohort [8]. In a cohort of patients hospitalized for any surgery, Gialdini et al. found an association between POAF and long-term ischemic stroke, both in unselected cardiac surgery patients (HR 1.3; 95% CI 1.1–1.6) and non-cardiac patients (HR 2.0; 95% CI 1.7–2.3; *P* < 0.001 for interaction) [8]. Within an unselected cardiac surgery cohort, CABG patients constitute a large and relatively uniform group, characterized by mutual risk factors, advanced coronary artery disease, and ischemic cardiomyopathy as primary cause of LV dysfunction [33]. Heart valve surgery, on the other hand, the second largest cardiac cohort, is in itself heterogeneous with diverse risk factors. Beside these two major groups, there are a non-negligible number of miscellaneous procedures [12]. Hence, without relevant sensitivity or interaction analyses accounting for the type of cardiac surgery, results should not be directly applied to CABG patients [8].

Prior to the current study, a relevant question was whether associations between POAF and outcome might also be confounded by the increased occurrence of AF during follow-up. To illuminate the mechanisms behind these associations, we took POAF, AF during follow-up, and interactions there between into account. Gialdini et al. chose to censor patients if and when post-discharge AF occurred [8]. But as patients with POAF can be expected to have more post-discharge AF [2–4] and AF is a known risk factor for ischemic stroke [7, 14] this approach may violate the assumption of non-informative censoring. We chose instead to include information about AF during follow-up as a time-updated covariate since that strategy allowed us to assume a controlled direct effect of POAF [34, 35].

Altogether, our study showed that POAF after CABG provides prognostic information regarding ischemic stroke that goes beyond the occurrence of AF during follow-up and that is also valid in a long-term perspective.

Still, the vast majority of POAF patients are subjected to irregular heart rhythm on numerous occasions, before and immediately after hospital discharge [36] and later during follow-up [2–4]. This underlines that from a clinical perspective, POAF cannot be separated from the associated episodes of AF and should not merely be considered as a short phase of AF. Instead the entire burden of AF must be recognized. This should be taken into account when associations between POAF and outcome are addressed.

Regarding corresponding mechanisms reported in primary AF, it is known that irregular heart rhythm activates the coagulation cascade and induces vessel wall injury [15]. Hypothetically, irregular heart rhythm in proximity to open-heart surgery (POAF) [20, 21] can also result in increased coagulation and, at least partly, contribute to the occurrence of cerebral thromboembolic events. This speculation, if proven, would be clinically highly relevant since effective treatment is available [37]. In this context, our results support current guidelines to at least consider anticoagulation therapy for CABG patients with POAF [37]. Direct oral anticoagulants have evolved as a valid alternative to warfarin in AF patients, that also reduce the risk of new cardiovascular events in patients with atherosclerotic disease and may, therefore, be a better choice than warfarin for CABG patients with POAF [38]. However, POAF should primarily be viewed as a marker of increased ischemic stroke risk until causality is proven.

The current study shows an association between POAF and long-term risk of heart failure, after adjusting for AF during follow-up. This has not been previously established [4]. The interaction analysis revealed that the association between POAF and HF during follow-up was different in patients with and without recurrent AF.

Mechanistically, AF is a common cause of tachycardia-induced cardiomyopathy, and neurohormonal and structural changes in heart failure can in turn trigger and maintain AF [16, 17], but overall it is not possible to specify which condition causes which. It has been suggested that AF and heart failure are different expressions of the same underlying systemic disease, and inflammation is significantly involved in both conditions [16–19].

In short, AF and HF are closely interrelated. As opposed to the association between AF, coagulation and stroke, we are unable to present a probable mechanism that could account for increased heart failure in POAF patients. Instead POAF may just as well constitute a marker of a more or less subtle myocardial injury.

This study confirms an association between POAF and overall mortality [5]. Regarding potential mechanism for this association, the observed increase in overall mortality associated with POAF was largely represented by differences in cerebrovascular and cardiac mortality. Moreover, POAF gave no additional information regarding overall mortality after taking POAF-associated morbidities into account. This suggests that the increased mortality associated with POAF is partially driven by the later occurrence of ischemic stroke and heart failure. POAF was also specifically associated with cardiac and cerebrovascular mortality, as suggested by previous studies [2, 6].

From a clinical perspective, in all situations discussed above, POAF cannot be separated from the associated increased recurrence of AF and should not merely be considered as a short phase of AF. This should be taken into account when associations between POAF and outcome are addressed. Regardless, POAF provides important prognostic information early, before hospital discharge, which increases the opportunity to initiate potential treatment before the occurrence of associated complications.

## Limitations

Residual confounding could have influenced the results, even though the adjusted Cox models included many known risk factors for each outcome event.

Regarding the development of POAF after cardiac surgery there are proposed causative mechanisms, including increased systemic inflammation associated with ECC [9, 10]. This study is the first to show an increased incidence of AF for CABG patients compared with matched controls, regardless of POAF status, during the first year after surgery. Acquired information points to possible causality, i.e. that cardiac surgery to some extent may cause AF. But, overall, our observational study design does not permit any conclusions to be drawn regarding causal relationships between the CABG procedure and subsequent POAF and the observed outcome, and this constitutes a major limitation.

Overall, and specifically beyond the first postoperative year*,* only patients with POAF had increased occurrence of AF, both compared with matched controls and with non-POAF patients. Thus, this study adds to existing data that support an association between POAF and recurrent AF. However, it does not contribute to a clarification regarding causality, i.e. if POAF could be avoided, would that lead to a reduced incidence of AF and associated complications? This question remains to be answered which constitutes a limitation.

Contrary to primary AF, there are no established mechanisms behind the associations between POAF and cardiovascular outcome, nor does the current study add to that knowledge. Until such mechanisms are proven, POAF should merely be considered as a marker for an increased long-term cardiovascular risk.

The generalizability is limited to the CABG population. However, this is a large patient group in which anticoagulation is not routinely indicated, with a POAF incidence of 30%, which in turn is associated with a 25% relative risk increase for ischemic stroke. In that context, our findings are novel, valid, and truly generalizable to the CABG cohort, which was our aim.

The lack of data on medication during follow-up is a major limitation, especially the use of anticoagulation therapy that could have influenced the ischemic stroke risk. Based on current guidelines [37], prescription of oral anticoagulants was not routine, and previous results show a low prescription rate to patients with POAF [36]. In contrast, a high proportion of patients with primary AF in Sweden are prescribed anticoagulation therapy [39], which could have influenced, i.e. lowered, the incidence of ischemic stroke in patients with AF during follow-up [40].

## Conclusions

An association between POAF and AF during follow-up was confirmed, and only patients with POAF had an increased long-term AF occurrence. Absence of POAF was reassuring, with 95% of non-POAF patients remaining free of AF at 10 years and 92% at 15 years, no less than corresponding figures for matched controls (94% and 91%, respectively). Further, POAF was associated with long-term ischemic stroke, heart failure and corresponding mortality after CABG, even after adjustment for AF during follow-up. The morbidities associated with POAF–AF, ischemic stroke, and heart failure—contributed to the increased long-term mortality associated with POAF after CABG.

AF that occurs early after CABG surgery should not be considered an isolated phenomenon related to perioperative factors, but as a marker of an underlying pervasive cardiovascular dysfunction and an increased risk of AF long-term.

## Electronic supplementary material

Below is the link to the electronic supplementary material.

Supplementary file1 (DOCX 22 kb)
