# Supplementary material for: Compared with matched controls, patients with postoperative atrial fibrillation (POAF) have increased long-term AF after CABG, and POAF is further associated with increased ischemic stroke, heart failure and mortality even after adjustment for AF
Source: Clin Res Cardiol. 2020 Feb 8;109(10):1232–42. doi: 10.1007/s00392-020-01614-z (PMC7515855; doi:10.1007/s00392-020-01614-z)
Supplement: Supplementary file 1 — Supplementary file1 (DOCX 22 kb) [file 392_2020_1614_MOESM1_ESM.docx]

**Supplementary material**

**Compared with matched controls, patients with postoperative atrial fibrillation (POAF) have increased long-term AF after CABG, and POAF is further associated with increased ischemic stroke, heart failure and mortality even after adjustment for AF**

Clinical Research in Cardiology

Emma Thorén, Mona-Lisa Wernroth, Christina Christersson, Karl-Henrik Grinnemo, Lena Jidéus, and Elisabeth Ståhle

**Corresponding author:** Emma Thorén, M.D., Ph.D., Department of Surgical Sciences, Cardiothoracic Surgery, Uppsala University, Uppsala, Sweden;

E-mail address: emma.thoren@akademiska.se

| Supplementary Table 1 Classification of medical history^a^ | | |
| --- | --- | --- |
| Medical history | ICD-9 1996 | ICD-10 1997-2012 |
| Atrial fibrillation | 427D | I48 |
| Ischemic stroke | 433, 434, 435, 436 | I63, I64 |
| Heart failure | 428 | I50 |
| Non-cerebral thromboembolism | 415B, 444, 451, 452, 453, 557A | G45, H34, I26, I65, I66, I74, I81, I82, N280, O882 |
| Hemorrhagic stroke | 430, 431, 432 | I60, I61, I62 |
| Non-cerebral bleeding | 423A, 456A, 530H, 531A, 531C, 531E, 531G, 532A, 532C, 532E, 532G, 533A, 533C, 533E, 533G, 534A, 534C, 534E, 534G, 569D, 578A, 578B, 578X, 599H, 719B, 784H, 784W, 786D | D62, D683, H356, H431, H450, I230, I312, I850, I983, K226, K250, K252, K254, K256, K260, K262, K264, K266, K270, K272, K274, K276, K280, K282, K284, K286, K290, K625, K661, K920, K921, K922, M250, N02, N92, N938, N939, N950, R04, R31, R58 |
| Diabetes | 250 | E10, E11, E12, E13, E14 |
| *ICD* International classification of diseases, Ninth or Tenth Revision  ^a^ Primary or secondary diagnosis | | |

| Supplementary Table 2 Classification of outcome events^a^ | | |
| --- | --- | --- |
| Outcome event | ICD-9 1996 | ICD-10 1997-2012 |
| Atrial fibrillation | 427D | I48 |
| Ischemic stroke | 433, 434, 435, 436 | I63, I64 |
| Heart failure | 428 | I50 |
| Non-cerebral thromboembolism | 415B, 444, 451, 452, 453, 557A | G45, H34, I26, I65, I66, I74, I81, I82, N280, O882 |
| Hemorrhagic stroke | 430, 431, 432 | I60, I61, I62 |
| Non-cerebral bleeding | 423A, 456A, 530H, 531A, 531C, 531E, 531G, 532A, 532C, 532E, 532G, 533A, 533C, 533E, 533G, 534A, 534C, 534E, 534G, 569D, 578A, 578B, 578X, 599H, 719B, 784H, 784W, 786D | D62, D683, H356, H431, H450, I230, I312, I850, I983, K226, K250, K252, K254, K256, K260, K262, K264, K266, K270, K272, K274, K276, K280, K282, K284, K286, K290, K625, K661, K920, K921, K922, M250, N02, N92, N938, N939, N950, R04, R31, R58 |
| Overall mortality | All diagnoses | All diagnoses |
| Cardiac mortality | 394, 395, 396, 397, 398, 401, 402, 404, 411, 412, 413, 414, 420, 421, 422, 423, 424, 425, 426, 427, 428, 429 | I05, I06, I07, I08, I09, I10, I11, I13, I20, I21, I22, I23, I24, I25, I30, I31, I32, I33, I34, I35, I36, I37, I38, I39, I40, I41, I42, I43, I44, I45, I46, I47, I48, I49, I50, I51, I52 |
| Cerebrovascular mortality | 430, 431, 432, 433, 434, 435, 436, 437, 438 | I60, I61, I62, I63, I64, I65, I66, I67, I68, I69 |
| *ICD* International classification of diseases, Ninth or Tenth Revision  ^a^ Primary diagnosis or underlying cause of death | | |

| **Supplementary Table 3** Interactions between POAF and postoperative course regarding morbidity events | | | | | | | | | | |
| --- | --- | --- | --- | --- | --- | --- | --- | --- | --- | --- |
|  |  | Atrial fibrillation | | | Ischemic stroke | | | Heart failure | | |
| Interaction | No. | No. of events | HR (95% CI)^a^ | *P* value^b^ | No. of events | HR (95% CI)^a^ | *P* value^b^ | No. of events | HR (95% CI)^a^ | *P* value^b^ |
| POAF and postoperative course^c^ |  |  |  | 0.97 |  |  | 0.26 |  |  | 0.57 |
| Uncomplicated course | 5695 | 496 | 3.19 (2.66; 3.82) |  | 580 | 1.27 (1.07; 1.51) |  | 745 | 1.44 (1.24; 1.68) |  |
| Complicated course | 1450 | 139 | 3.21 (2.28; 4.53) |  | 192 | 1.05 (0.78; 1.40) |  | 315 | 1.34 (1.06; 1.68) |  |
| *HR* hazard ratio, *POAF* postoperative atrial fibrillation  ^a^ Hazard ratios are adjusted for age, gender, hypertension, history of ischemic stroke, heart failure, non-cerebral thromboembolism, hemorrhagic stroke, non-cerebral bleeding and diabetes, number of diseased coronary vessels, left main stenosis, left ventricular function, time of surgery, use of the internal mammary artery, and aortic cross-clamp time  ^b^ *P* Values for interaction  ^c^ An uncomplicated course was defined as no sternal wound infection, stroke, heart failure, myocardial infarction, reoperation, or renal dysfunction in relation to the index surgery | | | | | | | | | | |

| **Supplementary Table 4** Interactions between POAF and postoperative course regarding mortality | | | | | | | | | | |
| --- | --- | --- | --- | --- | --- | --- | --- | --- | --- | --- |
|  |  | Overall mortality | | | Cardiac mortality | | | Cerebrovascular mortality | | |
| Interaction | No. | No. of events | HR (95% CI)^a^ | *P* value^b^ | No. of events | HR (95% CI)^a^ | *P* value^b^ | No. of events | HR (95% CI)^a^ | *P* value^b^ |
| POAF and postoperative course^c^ |  |  |  | 0.88 |  |  | 0.57 |  |  | 0.45 |
| Uncomplicated course | 5695 | 1808 | 1.19 (1.07; 1.31) |  | 709 | 1.35 (1.16; 1.58) |  | 154 | 1.61 (1.17; 2.22) |  |
| Complicated course | 1450 | 648 | 1.20 (1.03; 1.41) |  | 287 | 1.25 (0.98; 1.58) |  | 64 | 1.28 (0.78; 2.12) |  |
| *HR* hazard ratio, *POAF* postoperative atrial fibrillation  ^a^ Hazard ratios are adjusted for age, gender, hypertension, history of ischemic stroke, heart failure, non-cerebral thromboembolism, hemorrhagic stroke, non-cerebral bleeding and diabetes, number of diseased coronary vessels, left main stenosis, left ventricular function, time of surgery, use of the internal mammary artery, and aortic cross-clamp time  ^b^ *P* Values for interaction  ^c^ An uncomplicated course was defined as no sternal wound infection, stroke, heart failure, myocardial infarction, reoperation, or renal dysfunction in relation to the index surgery | | | | | | | | | | |
